# Kin selection and the evolution of plant reproductive traits

**DOI:** 10.1098/rspb.2016.0789

**Published:** 2016-11-16

**Authors:** Kamaljit S. Bawa

**Affiliations:** 1 Department of Biology, University of Massachusetts, Boston, MA 02125-3393, USA; 2 Ashoka Trust for Research in Ecology and the Environment, Bangalore, Karnataka 560064, India

**Keywords:** kin selection, placentation, pollen aggregations, pollination modes, seed number

## Abstract

Competition among developing seeds and sibling rivalry within multiovulated ovaries can be deleterious for both the maternal parent and the siblings. Increased genetic relatedness of seeds within the ovary may foster kin selection and reduce the deleterious consequences of sibling competition. The pollen parent may also be selected for siring all progeny within a fruit. I propose a series of hypotheses to explain the evolution of a number of reproductive traits in angiosperms in the context of kin selection and sibling rivalry within the ovaries of angiosperms. I present evidence to show that a single-pollen parent, indeed, often sires seeds within multiovulated ovaries. Various types of pollen aggregations and transfer of such pollen masses to the stigmas of flowers by specialized pollinators make this increased genetic relatedness possible. An alternative mode to reduce sibling rivalry may be the reduction of ovule number to one, an evolutionary trend that has independently occurred many times in flowering plants. Finally, I build on previously established correlations to predict two sets of correlations among reproductive traits. In the first case, large showy flowers, transfer of pollen *en masse* by specialized pollinators, and multiovulated ovaries and multisided fruits seem to be correlated. In the second case, the previously established correlations among small and inconspicuous flowers, pollination by wind, water or generalist insects, flowers and fruits with few or single ovules and seeds, respectively, may also include monoecy or dioecy. Although correlations among many of these traits have been established in the past, I invoke kin selection and sibling competition to explain the evolution of correlated traits as two distinct evolutionary pathways in angiosperms.

## 1. Introduction

Plants vary enormously in the number of ovules within an ovary, the placentation that sustains ovules and the resulting embryos and seeds, pollination mechanisms, the parentage of pollen that may sire the ovules within an ovary and the number of embryos that may develop into seeds within fruits. In a landmark paper, Kress [1] examined the evolution of some of these traits within a single conceptual framework of kin selection developed by Hamilton [2]. Kress [1] argued that in multiovulated ovaries, sibling competition may foster the evolution of specialized pollination mechanisms to deliver pollen grains from one or few pollen parents to the stigma(s) of a flower. This could increase the genetic relatedness of developing offspring, thus fostering kin selection and reducing sibling competition that could be detrimental to the maternal parent. The attractiveness of this idea lies in its ability to explain the correlation between the evolution of multiovulated ovaries and pollen aggregations (see also Harder & Johnson [3]).

Here, I extend the notion of kin selection and sibling rivalry within the ovaries of angiosperms to explain the evolution of a number of reproductive traits in angiosperms. Intense competition among developing seeds for maternal provisioning of resources and sibling rivalry within multiovulated ovaries can be deleterious for both the maternal parent and the siblings. However, if the developing seeds are full sibs, then there may be opportunities for kin selection and cooperation. Evidence for kin recognition and cooperation in plants is gradually accumulating [4–6], though except for Kress [1], the idea has not been applied to ovaries, an obvious place where opportunities for cooperation and competition prevail.

There may also be selection for full sibship in the paternal genome, because sibling competition among developing embryos or seeds may also be detrimental to the paternal parent. As in the case of many animals, especially insects [7], males may have evolved strategies to sire all progeny within an ovary. Thus, both maternal and paternal parents may favour full sibship of seeds within an ovary. I show that paternity analyses of seeds within single fruits, indeed, indicate that a single parent often sires seeds in multiseeded fruits.

Fertilizing all ovules within a fruit by a pollen parent may require aggregated pollen and transfer of these aggregations by specialized pollinators as pointed out by Kress [1]. An alternative strategy might be to reduce the number of ovules to one per ovary that may require for pollination just one or few pollen grains transported by generalist pollinators, wind or water.

Finally, on the basis of kin selection and sibling rivalry, I show that the evolution of reproductive traits in flowering plants may have followed two broad trends. One may be represented by species that have large and showy flowers, specialized modes of pollination, pollen in aggregated units, multiovulated ovaries and many-seeded fruits in which one pollen parent often sires all seeds. The other may be species with small and inconspicuous flowers, often unisexual, with a generalist mode of pollination, including water and wind pollination, and single or few ovules yielding single or few seeded fruits. To the best of my knowledge, such trends and correlations have not been explained before in the context of sibling competition and kin selection within ovaries.

My hypothesis links selection for increased genetic relatedness of seeds in multiovulate ovaries with specialized modes of pollination and conspicuous floral displays on the one hand, and the evolution of reduction in ovule and seed number with evolution of a generalist mode of pollination and small inconspicuous flowers, on the other hand. It consists of the following three elements.

(a) *Increased genetic relatedness of seeds within an ovary.* In species with multiovulate ovaries, there has been a trend towards increased genetic relatedness of seeds within a fruit involving pollen aggregations and simultaneous arrival of multiple pollen grains from one pollen donor to the stigma.

(b) *Specialized modes of pollination and floral displays.* Pollination by specialized vectors that can transfer pollen *en masse* from a single-pollen parent has fostered pollen aggregations and synchronous deposition of pollen from one donor.

(c) *Reduction in the number of ovules or seeds.* Concurrent with trends described in (*a*) and (*b*), reduction in the number of ovules or seeds within fruits was accompanied by the evolution of a generalist mode of pollination.

In the following, I explore these hypotheses and their predictions as well as comparative evidence or trends to test the predictions.

## 2. Increased genetic relatedness of seeds within the ovary

The evolution of a number of traits that increase the genetic relatedness of developing embryos can be explained in the context of kin selection. These traits include multiple pollen units and synchronous arrival of pollen grains on the stigma. Another major trait, the specialized mode of pollination, is discussed in a subsequent section.

### (a) Pollen units

#### (i) Background

Kress [1] was among the first to suggest that in many flowering plants, pollen grains are aggregated into packets so that, when transferred *en masse* to the stigmas of a flower and followed by successful fertilization, they increase the genetic relatedness of resulting embryos. He further showed a positive correlation between number of pollen grains in pollen units and the number of ovules in the ovary. Such correlations have been subsequently reported within the same genus, for example in *Acacia,* a mimosoid legume [8,9]. Moreover, the stigma morphology in the same group of legumes is such that only one polyad, with the number of pollen grains equal to or exceeding the number of ovules, may fit in the stigma cavity [10–12]. When two or more polyads are found in the stigmatic cavity, only one is properly oriented to send the pollen tubes to the ovary [12].

#### (ii) Hypothesis

Pollen units (polyads, pollinia and other pollen assemblages) have evolved to increase the genetic relatedness of seeds within the developing ovary.

#### (iii) Evidence

In *Acacia myrtifolia*, 52% of the flowers are pollinated by just one polyad, 5% by more than one polyad and 43% are without pollen [13]. Genetic analyses reveal that sibship of seeds within fruits of *Acacia melanoxylon* is one in one population and 0.63 in another population [14]. In some cases, for example, in *Acacia aroma* and *Acacia micrantha,* the whole progeny arrays have a higher probability of being full rather than half-sibs [11] . In *Parkia biglobosa,* where the polyads have 16 or 32 pollen grains, one pollen parent sires all the 24 or so seeds within a fruit [15].

Pollen aggregations attain their ultimate unity and complexity in the Orchidaceae, arguably one of the most advanced families of flowering plants, in which all pollen grains of a flower are united in a pollinium. Orchid flowers also have thousands of ovules within the ovary, setting up intense competition among developing seeds. This competition may be reduced in two ways. One is by increasing the genetic relatedness of developing seeds by having all seeds sired by a single-pollen parent. The other is by reducing the development time for the embryos by delayed fertilization that is known to occur in some orchids. Delayed fertilization, however, appears to be more common in wind-pollinated species (see table 1 in Sogo & Tobe [16]), owing to reasons that I explain later.

Pollination by single polyads or pollinia is not universal. In Asclepideaceae, more than one pollinium typically pollinates a flower. Nevertheless, paternity analyses of fruits in *Asclepias syriaca* revealed that a single-pollen parent sired seeds in each fruit that was analysed [17]. In *A. exaltata,* seeds in 85% of fruits were found to have single-pollen parents [18].

Full sibship within a fruit is not contingent upon pollen aggregations. In the syconium of a fig, the hundreds of tiny flowers are uniovulate, but because of the tight packing of flowers, the inside of a syconium may be regarded as equivalent to a multiovulate ovary. Generally, a single female wasp carrying pollen from one pollen donor fertilizes all the flowers in a syconium. Thus, the developing seeds in close proximity to each other are essentially full sibs. Figs represent an extreme example of specialized pollination. In contrast, the orchids, with multiovulate ovaries, as discussed in the subsequent section, rely on a very different pollination mechanism to attain full sibship of seeds within a fruit.

Although polyads and pollinia are the most visible manifestations of pollen aggregations, pollen in animal-pollinated species can occur as clumps of many pollen grains. First, in animal-pollinated species, pollen is coated with pollenkit that makes pollen adhere to the body of the pollinators, and also to other pollen. Second, in many species, pollen grains are loosely held together by structures originating from the pollenkit. In Onagraceae, viscin threads hold pollen in the form of loose aggregations and in Fabaceae, ‘exinal connections’ exist among pollen grains [19].

### (b) Synchronous arrival of pollen

#### (i) Background

A wide variety of mechanisms allow the simultaneous arrival of many pollen grains from the same pollen parent in multiovulated species. Once a certain number of pollen grains have landed on the stigma, the deposited grains may interfere with the fertilization of ovules by pollen grains subsequently landing on the stigma. Interference could be mechanical or it could occur via the fertilization of ovules by pollen grains that are first deposited on the stigma.

Once a large number of pollen grains arrives on the stigma, further pollen deposition and/or fertilization by late-arriving pollen may be limited in a variety of ways. As mentioned earlier, in many legumes with polyads, the stigmatic cavity can hold only one polyad in correct orientation. In various species of *Tababeuia* and other genera in the Bignoniaceae, the bifid stigmatic lobes close immediately after the pollen is deposited. It is uncertain if the stigmatic lobes subsequently open in these 1-day flowers. Nevertheless, even if they do, the early-arriving pollen may already have germinated and occupied the stylar canal by the time more pollen lands on the stigma. Unfortunately, rates at which pollen lands on stigmas are poorly documented. Closure of the stigma following pollen deposition also enhances male fitness by ensuring that all seeds sired in the ovary will be from the same pollen parent.

The effectiveness of late-arriving pollen grains can also be pre-empted in the absence of morphological features. Pollen germinates within a few minutes after it lands on the stigma [20], so the late-arriving pollen grain would have a strong competitive disadvantage in gaining access to ovules.

#### (ii) Hypothesis

In multiovulated species, selection should reduce asynchrony in the arrival of pollen on the stigma and favour evolution of mechanisms that promote simultaneous deposition of many grains from a single-pollen parent that lead to full sibship among developing seeds.

#### (iii) Evidence

Data are scarce to test this hypothesis. Indeed, there are species in which pollen on the stigma is derived from a single-pollen parent, as for example in species in families such as the Bignoniaceae described above. However, it is also a common observation that flowers receive multiple visits from pollen parents. Genetic analyses of progeny from single fruits offer a relatively easy way to test the hypothesis.

Much of the genetic evidence so far indicates that in a majority of cases, a single-parent sires seeds within a fruit in multiovulate species. I have already cited evidence for several species of Acacia. In *Tabebuia rosea-alba*, referred to earlier, paternity analyses revealed that seeds in many fruits were full sibs, and the number of pollen donors to a flower ranged from 1.21 to 1.50 [21]. Similarly, in *Theobroma cacao*, the number of pollen donors to a flower was 1.65 when compared with 10.1 for the whole tree [22]. However, such results are not universal. Seeds within fruits of wild radish [23], *Mimulus ringens*, and *Ipomopsis aggregata,* have multiple paternity [24].

Apparently, pollen aggregations as well as synchronous arrival of pollen grains from one donor on the stigma and pollen carryover, respectively, are important factors in determining single versus multiple paternity of seeds within fruits [3,24]. If kin selection has played an important role in determining the seed numbers within a fruit, further genetic analyses will reveal that seeds in multiovulate angiosperms are more likely to be full rather than a mixture of full and half-sibs. Finally, full sibship of seeds within fruits owing to polyads and pollen aggregation and synchronous deposition of pollen from the same parent, as pointed out in the beginning, can also be explained in terms of selection for male fitness and pre-emption of other males for siring the ovules in the same ovary.

## 3. Modes of pollination and floral displays

### (a) Background

Flowering plants have an array of pollination systems. Following Ashworth *et al*. [25], I define specialist pollinators as pollinators ‘which depend exclusively on one or a few plant taxa as food sources’ and generalist pollinators as pollinators ‘which are able to feed on a wide array of flower species’. Specialist pollinators show close evolutionary relationships with the specific plant species and respond to similar phenological cues [26,27].

Transfer of aggregated pollen in the form of polyads, pollinia, pollinaria or pollen held together by viscin threads or other means, and simultaneous arrival of many pollen grains from a single donor on the stigma, require adherence and removal of pollen from specific parts of pollinators that at any given time forage among individuals of a single species.

### (b) Hypothesis 1

Thus, it is logical to predict that specialist pollinators are more likely to transfer pollen *en masse* in multiovulate species than the generalist pollinators, and that such species are also more likely to have many-seeded fruits than species pollinated by generalist pollinators. Willson [28] also predicted an association between pollen aggregations and specialist pollinators, but in the context of sexual selection.

### (c) Evidence

Indeed, pollen aggregations are associated with pollination by specialist pollinators [3,29]. Furthermore, species pollinated by wind, independent of phylogenetic constraints, often have few or one ovule(s) and single-seeded fruits [1,30,31]. There is a need to map the first set of correlations on phylogenies as well to further elucidate the evolutionary basis. Such analyses are underway for monocotyledons [32].

### (d) Hypothesis 2

I also predict that species pollinated by generalist insects will have single-seeded fruits, because stigmas of such flowers, as in the case of wind-pollinated species, are likely to receive pollen from a diverse array of pollen donors, thus setting up intense competition among developing embryos if the fruits are many-seeded or if many ovules within the ovary are fertilized.

### (e) Evidence

There is indirect evidence for correlation between the generalist mode of pollination and single-seeded fruits. For example, dioecy in flowering plants is correlated with generalist pollination systems and seed dispersal by specialized frugivores, which generally prefer few- or single-seeded fruits [33]. Intrasexual competition may be more pronounced in dioecious species, and made more intense by generalist pollination modes that deliver pollen from many pollen parents onto the stigma of a single flower, leading to strong selection for the reduction of sib competition within the ovary and thereby resulting in ovaries with few or one ovule, or few- or single-seeded fruits. If these assumptions are upheld, then sib competition may also explain the association between single or few seeded fruits and dioecy.

In summary, what is noteworthy here are the new postulated relationships between specialized pollination modes, multiovulate ovaries, many-seeded fruits and full sib-ship within fruits on the one hand, and generalist mode of pollination and single or few seeded fruits on the other hand.

A correlation between flower size and pollinator specialization may also exist. Flowers of dioecious species pollinated by generalist insects are smaller than species with hermaphrodite flowers, many of which are pollinated by specialist pollinators [34]. Many species pollinated by specialists produce pollen aggregations that are characteristic of multiovulate species. There are no data on the relationship between flower size and ovule or seed number, but I predict the two traits to be positively correlated.

## 4. Reduction in ovule and seed number

### (a) Background

A decrease in the number of developing seeds within fruits should minimize sibling rivalry. In species with single ovules, there is no potential for kin selection within the ovary. Correspondingly, in multiovulate species, there is perhaps intense sibling competition. As argued above, increased genetic relatedness could reduce the deleterious consequences of such competition. Thus, kin selection should strongly affect the number of seeds within the ovary, and genetic relatedness of seeds within the ovary.

### (b) Hypothesis

Evolution of seed number has proceeded in two directions: one is towards reduction in seed number to one within the ovary, and the other is towards many-seeded fruits sired by a single-pollen parent.

### (c) Evidence

One prediction of the hypothesis is that seed number distribution in flowering plants should be bimodal—most species with either single-seeded fruits or many-seeded fruits. Indeed, Ganeshaiah & Shaanker [30] showed that in a large sample of flowering plants, such a distribution pattern prevails. There has been evolution towards a reduction in the number of ovules, and uniovulate flowers have independently evolved several times in angiosperms [35]. Decreasing the number of ovules and thereby decreasing the number of developing seeds can certainly reduce competition among siblings. Reduction in seed number, however, can also occur without reduction in ovule number. In many plants with multiovulate flowers, only one of the many ovules develops into seeds [36]. In most of these cases, abortion of ovules or embryos occurs soon after fertilization.

Furthermore, as discussed above, in the ovaries of wind-pollinated flowers or flowers pollinated by generalist insects, intense competition among sibs sired by different pollen parents should result in evolution towards decreased seed number culminating in single-seeded fruits. Indeed, there is correlation between generalist pollination modes, including wind pollination and single- or few-seeded fruits [1,37,38]. Conversely, multiseeded fruits and multiovulate ovaries are associated with flowers pollinated by specialized pollinators (see Kress [1]).

Reduction in seed number per fruit can also be influenced by seed size, which, in turn, is affected by life-history traits and habit [39]. In grasses, reproductive uncertainty owing to wind pollination has been implicated in a decrease in ovule number [38]. Other explanations for reduction in ovule number include advantages that might accrue to wind-pollinated species from packaging fewer ovules in many flowers to capture pollen from a larger area than fewer flowers with many ovules in each [31]. However, ovaries with few or one ovule also occur in many animal-pollinated species.

Seed number is also influenced by the mode of dispersal. Among wind-dispersed taxa, many-seeded fruits are characteristic of species with seeds as units of dispersal and single-seeded fruits typically occur in species with fruit as the unit of dispersal [30]. Seed number is just one example of reproductive characters considered above that are under multiple selective forces.

Although the reduction in ovule and seed number can be explained by multiple factors, the correlations among flower size, floral sexuality, mode of pollination and ovule number can only be well explained in the context of kin selection. If paternity analyses of two- to few-seeded fruits in species with small unisexual flowers pollinated by generalist pollinators reveal that they are less likely to be full sibs when compared with multiseeded fruits from large bisexual flowers pollinated by specialist pollinators, the role of sib competition in driving seed number can receive further credence. Furthermore, full sibs within fruits may show less variance in seed size and weight than half-sibs.

## 5. Concluding remarks

Apart from the correlations outlined above, it is possible that sibling competition may also explain some trends in the evolution of the gynoecium, particularly placentation. Little has been written about the evolution of placentation since Puri's classic 1952 paper [40] nearly 65 years ago. The last comprehensive account was by Stebbins [35], almost 40 years ago. Both Puri and Stebbins traced the evolution of placentation and commented on drivers underlying the changes in terms of evolutionary trends associated with morphology, anatomy and resource utilization. In the evolution of placentation, multiple pathways lead to reduction in ovule number to one, and this is consistent with the idea that sibling competition could reduce ovule number, especially in species in which flowers are likely to receive pollen from multiple parents, as discussed above.

Thus, sib competition within the ovary may have driven the evolution of many traits associated with the gynoecium, including placentation, pollen aggregations, modes of pollination including floral displays, and ovule and seed number. Indeed, there are a number of other factors, such as habit, pollinators and seed dispersal agents that also play an important role in the evolution of these traits. However, presently, only kin selection and sib competition explain the evolution of multiple correlated plant reproductive traits.

**Figure 1.**
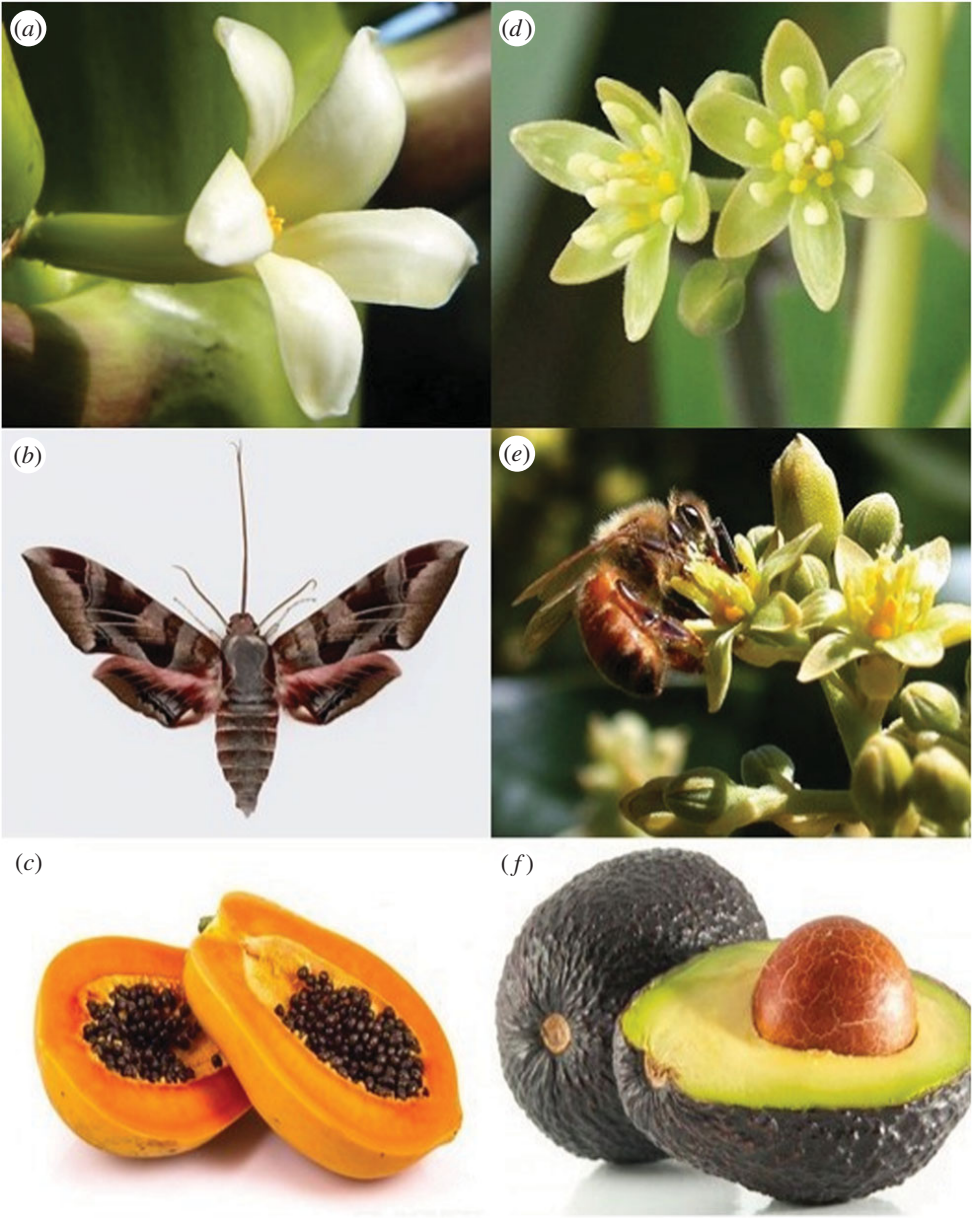
Two main evolutionary trends in the evolution of correlated reproductive traits: large flowers (*a*), specialized pollinators (*b*), ovaries or fruits with many ovules or seeds (*c*); small flowers (*d*), generalized pollinators (*e*) and ovules or fruits with few or one ovule(s) or seed(s) (*f*). Flower of *Carica papaya* (*a*), its pollinators, Sphingidae (*b*) and fruit (*c*). Flowers of *Persea americana* (*d*) its generalist pollinator species-honeybee (*Apis mellifera*) (*e*) and fruit (*f*). Source: Google Images on the World Wide Web.

As shown in table 1, it is possible that as angiosperms evolved and diversified, evolution of reproductive traits proceeded in two directions. In the first case, large showy flowers became associated with multiovulate ovaries and pollen aggregations evolved to reduce sib competition through kin selection. This pathway would have been fostered by large floral displays and pollination by specialist pollinators that could transfer pollen *en masse*, increasing the genetic relatedness of seeds within fruits (figure 1*a–c*). In the second case, small flowers became associated with uniovulate ovaries with pollination by wind, water or generalist insects (figure 1*d*–*f*). In woody species these traits also became associated with unisexual flowers (table 1). Correlated transformations in placentation, ovule number, pollen units, pollination modes, sexual systems and seed numbers may have occurred repeatedly. It is not clear if modes of pollination drove changes in ovule and seed numbers or vice versa.

**Table 1.** Postulated correlations driven by kin selection and sibling rivalry resulting in two distinct evolutionary trends.^a^

correlations	reference
*I large flowers, pollen aggregations, specialist pollinators, multiovulate ovaries*	
polyads, pollen aggregations, and multiovulate ovaries	[1]
polyads, pollen aggregations, and specialist pollinators	[28]
*large flowers*, polyads, pollen aggregations, specialist pollinators, *synchronous arrival of pollen grains, full sibship of seeds within fruits*, multiovulate ovaries or many-seeded fruits	present study^b^
*II small flowers, generalist pollinators, unisexual flowers, few or one ovules*	
monads and wind pollination	[1]
wind and water pollination and few or single ovules	[1,30]
small flowers, generalist mode of pollination, dioecy, tropical distribution, fleshy fruits with one or a few seeds	[37,41]
small inconspicuous flowers, abiotic pollination, many-flowered inflorescences, dioecy, tropical distribution, woody growth form and fleshy fruits	[42]
small flowers, generalist mode of pollination *including wind and water pollination*, *monoecy or dioecy*, *ovaries with few or one ovule(s)*	present study^b^

^a^Note that although many authors have shown several of the correlations listed above, only Kress [1] and Bawa (present study) invoke kin selection and sibling rivalry as the driving forces for the observed correlations.

^b^New traits involved in correlations are shown in italics.

We have preliminary evidence that many of the correlations postulated above hold for families within the monocotyledons. For example, more than 80% of families in diverse monocot lineages show two broad sets of correlations: (i) small flowers and one or few ovules or seeds and (ii) large flowers and many ovules or seeds [32].

Curiously, plant evolutionary biologists have considered the evolution of correlated traits in isolation, except of course the evolution of pollen units that has occasionally been linked with pollination mode [1,3,29]. I have argued that strategies in deployment and development of ovules, and pollination modes in which both the nature of pollen units and their arrival on the stigma play a critical role, may well shape the nature of competition, conflict and cooperation among developing embryos in the ovary and hence the seed number in fruits. Thus, our studies of plant evolution can be greatly enriched by reconsidering the evolution of reproductive traits in association with one another.

Consideration of kin selection and sibling competition may reveal further correlations and provide additional insights into the evolution of reproductive strategies in flowering plants. For example, there are size-related correlations among flowers, fruits and seeds [43]. Large flowers thus may be correlated with a whole suite of traits. Second, the correlation between generalist pollination and single- or few-ovulate ovaries may also involve dioecy, especially in woody species, because of the demonstrated correlation between dioecy and pollination by generalist insects on the one hand and between dioecy and single- or few-seeded fleshy fruits dispersed by specialized frugivores. Third, species pollinated by generalist pollinators or wind may have disproportionately higher representation among species with delayed fertilization, because the inflorescences in these species receive pollen from multiple donors and delayed fertilization can provide the maternal parent more time to assess the quality of the paternal parent.

Kin selection in plants has generally followed two lines of inquiry. First is the genetic relationship of the triploid endosperm with the diploid embryo and the effect of this relationship on the nutritional control and development of the seed by the maternal parent [44–46]. The second is the interaction among kin during the growth of seedlings [47–49]. In contrast, interactions among developing seeds within an ovary have received less attention (but see [36]). Yet, because of the proximity of siblings, the ovary is an obvious theatre of play for kin selection among the developing embryos. Close proximity also offers the opportunity for suggested mechanisms of kin recognition such as association, phenotype matching and recognition alleles [50] to operate often. Proponents of cooperation among plants [4] could seize this opportunity to gain new insights into ecology and evolution of plants.

In summary, I have argued that a suite of characters hierarchically mediates interactions within the ovary, starting from the placement of ovules (placentation) to the evolution of diverse modes of pollination, including pollen assemblages and transfer of pollen *en masse* that set the stage for kin selection within ovaries. I have outlined the ways to test the operation of kin selection (genetic relatedness of seeds within ovaries, and variation in abortion rates, seed size and seed weight). The direction of evolution in correlated traits, however, remains uncertain. Mapping of correlated traits on phylogenies offers the possibility of unravelling the direction and temporal dimensions of evolutionary changes in reproductive traits. We have some of these analyses underway [32].
